# μ-Cyanido-κ^2^ *C*:*N*-dicyanido-κ^2^ *C*-bis­(*N*-ethyl­ethylenedi­amine-κ^2^ *N*,*N*′)copper(II)copper(I)

**DOI:** 10.1107/S160053681400172X

**Published:** 2014-01-31

**Authors:** Peter W. R. Corfield, Joseph F. Michalski

**Affiliations:** a Department of Chemistry, Fordham University, 441 East Fordham Road, Bronx, NY 10458, USA

## Abstract

In the title complex, [Cu^I^Cu^II^(CN)_3_(C_4_H_12_N_2_)_2_], the Cu^I^ and Cu^II^ ions and a bridging cyanide group lie on a twofold rotation axis. The Cu^II^ ion is in a slightly-distorted square-pyramidal coordination environment, with the N atoms of the two symmetry-related *N*-ethyl­ethylenedi­amine ligands occupying the basal positions and an N-bonded cyanide group in the apical position. The Cu^I^ ion is in a trigonal-planar coordination environment, bonded to the C atom of the bridging cyanide group and to two terminal cyanide groups. In the crystal, N—H⋯N hydrogen bonds involving two of the symmetry-unique N—H groups of the *N*-ethyl­ethylenedi­amine ligands and the N atoms of the terminal cyanide ligands link the mol­ecules into strands along [010].

## Related literature   

The title compound was synthesized as part of our continuing study of structural motifs in mixed-valence copper cyanide complexes containing amine ligands. For descriptions of similar discrete mol­ecular copper cyanide complexes, see: Corfield *et al.* (2012 ▶); Pretsch *et al.* (2005 ▶); Pickardt *et al.* (1999 ▶); Yuge *et al.* (1998 ▶). For mixed-valence copper cyanide complexes crystallizing as self-assembled polymeric networks, from preparations similar to those used in the present work, see: Williams *et al.* (1972 ▶); Colacio *et al.* (2002 ▶); Kim *et al.* (2005 ▶), and also Corfield & Yang (2012 ▶), although this last one involves only Cu^II^ ions.
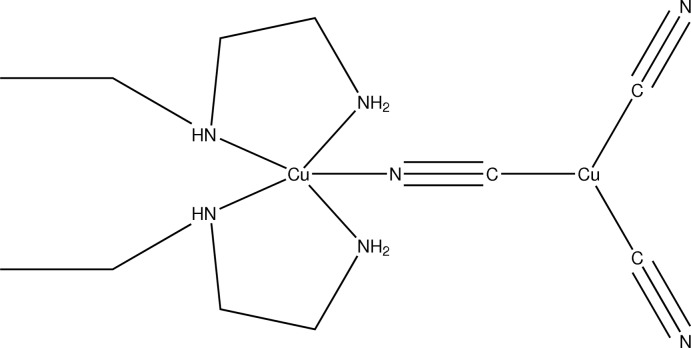


## Experimental   

### 

#### Crystal data  

[Cu_2_(CN)_3_(C_4_H_12_N_2_)_2_]
*M* *_r_* = 381.45
Monoclinic, 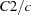

*a* = 11.425 (1) Å
*b* = 9.679 (2) Å
*c* = 15.205 (3) Å
β = 91.52 (1)°
*V* = 1680.8 (5) Å^3^
*Z* = 4
Mo *K*α radiation
μ = 2.53 mm^−1^
*T* = 301 K
0.33 × 0.30 × 0.30 mm

#### Data collection  

Enraf–Nonius CAD-4 diffractometer
Absorption correction: integration (Busing & Levy, 1957 ▶) *T*_min_ = 0.529, *T*_max_ = 0.587
3737 measured reflections
1835 independent reflections
1674 reflections with *I* > 2σ(*I*)
*R*_int_ = 0.020
3 standard reflections every 120 min intensity decay: 2.3 (6)%

#### Refinement  

*R*[*F* ^2^ > 2σ(*F* ^2^)] = 0.020
*wR*(*F* ^2^) = 0.062
*S* = 1.06
1835 reflections
103 parameters
H atoms treated by a mixture of independent and constrained refinement
Δρ_max_ = 0.22 e Å^−3^
Δρ_min_ = −0.25 e Å^−3^

### 

Data collection: *CAD-4 Software* (Enraf–Nonius, 1994) ▶; cell refinement: *CAD-4 Software*; data reduction: data reduction followed procedures in Corfield *et al.* (1973 ▶); data were averaged with a local version of *SORTAV* (Blessing, 1989 ▶); program(s) used to solve structure: *SHELXS97* (Sheldrick, 2008 ▶); program(s) used to refine structure: *SHELXL97* (Sheldrick, 2008 ▶); molecular graphics: *ORTEPIII* (Burnett & Johnson, 1996 ▶); software used to prepare material for publication: *SHELXL97*.

## Supplementary Material

Crystal structure: contains datablock(s) meed, I. DOI: 10.1107/S160053681400172X/lh5680sup1.cif

Structure factors: contains datablock(s) I. DOI: 10.1107/S160053681400172X/lh5680Isup2.hkl

CCDC reference:

Additional supporting information:  crystallographic information; 3D view; checkCIF report

## Figures and Tables

**Table 1:** Selected bond lengths (Å)

Cu1—C1	1.931 (3)
Cu1—C2	1.9406 (18)
Cu2—N3	2.0403 (14)
Cu2—N6	2.0456 (14)
Cu2—N1	2.142 (2)
C1—N1	1.139 (4)
C2—N2	1.136 (2)

**Table 2:** Hydrogen-bond geometry (Å, °)

*D*—H⋯*A*	*D*—H	H⋯*A*	*D*⋯*A*	*D*—H⋯*A*
N3—H3*B*⋯N2^i^	0.79 (2)	2.49 (2)	3.181 (2)	147 (2)
N6—H6⋯N2^ii^	0.81 (2)	2.34 (2)	3.112 (2)	160.9 (17)

Symmetry codes: (i) 
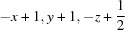
; (ii) 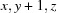.

**Fig. 1.**
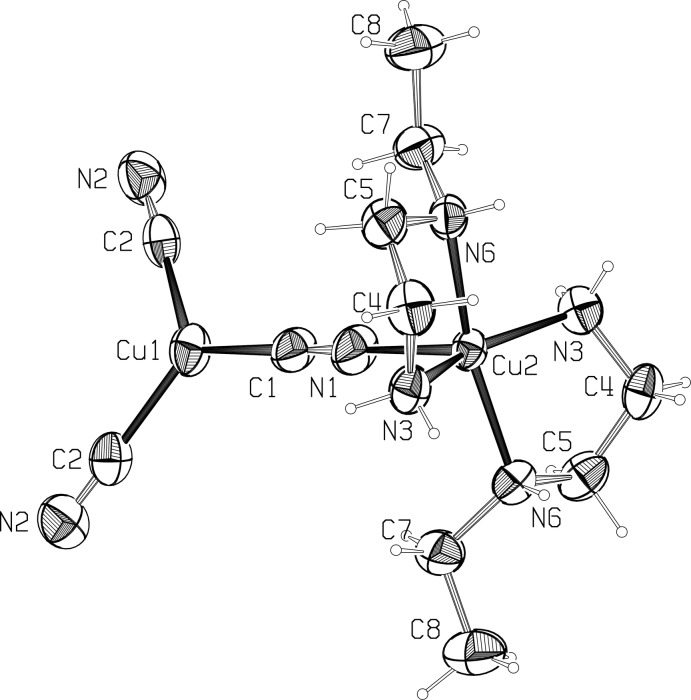
The molecular structure of the title molecule, with ellipsoids at the 50% level. Atoms with the same labels are related by the two- fold axis at 1/2, y, 1/4.

**Fig. 2.**
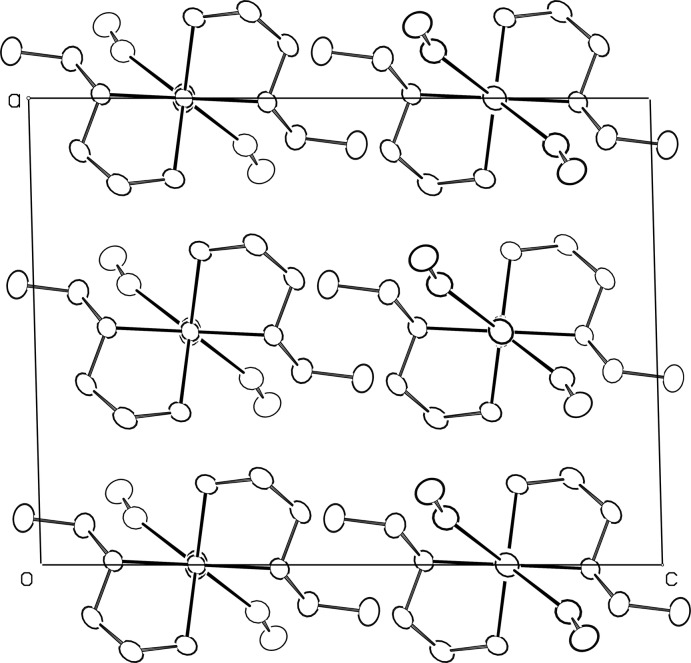
Packing of the title complex, viewed along the b axis. Ellipsoid outlines at 30% probability.

**Fig. 3.**
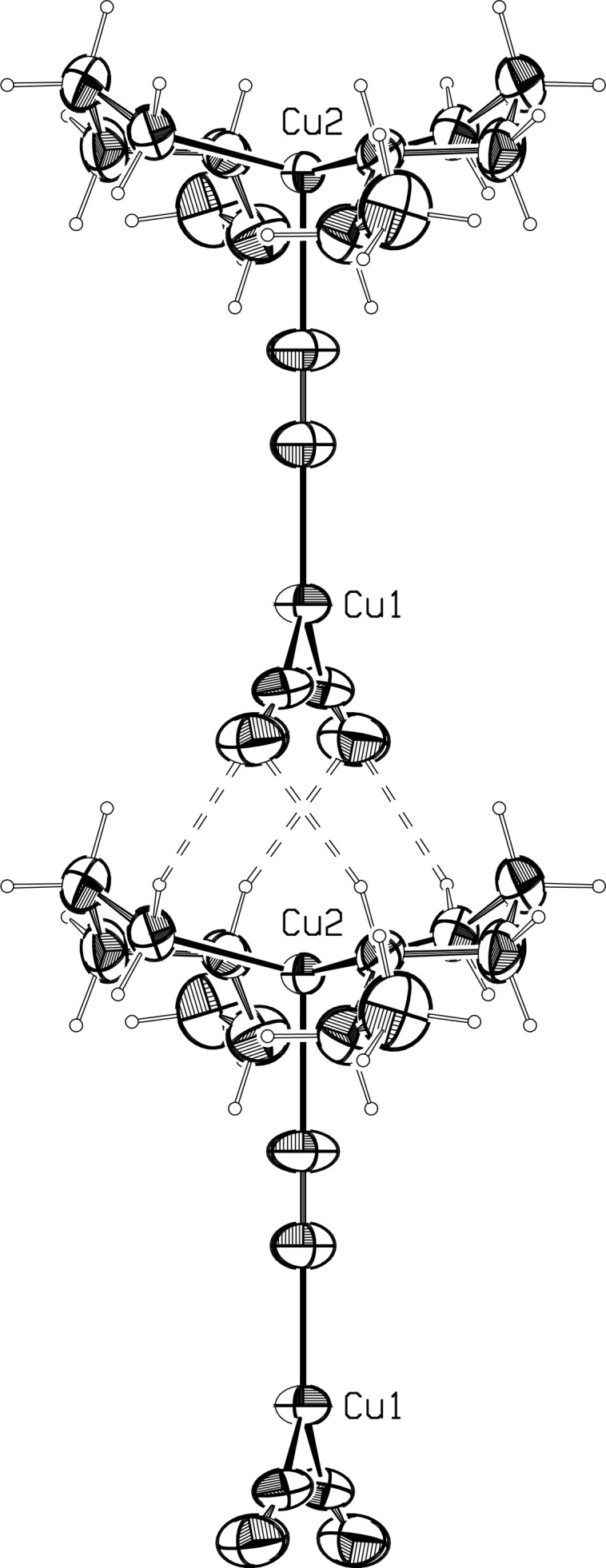
View of the title complex perpendicular to the crystallographic twofold axis, indicating molecules connected by hydrogen bonds, which are shown as dashed lines. Ellipsoids at 50% probability.

## supplementary crystallographic information

### 1. Results and discussion

The structure determination of the title compound was undertaken as part of a continuing study of mixed-valence copper cyanide complexes containing amine ligands, with the goal of learning how to direct synthesis of specific polymeric structures. In these compounds, the divalent copper atoms are stabilized by the coordinated amines against reduction by the cyanide groups. In the present work, the synthesis involved the bidentate base *N*-ethyl­ethylenedi­amine (eten), under conditions expected to produce a polymeric structure, as in Williams *et al.* (1972) or Colacio *et al.* (2002). The crystal structure is made up of discrete molecules, as shown in Fig. 1, with terminal cyanide groups that are not involved in covalent polymeric linkages, and is similar to structures previously reported by us (Corfield *et al.*, 2012) or by others (Yuge *et al.*, 1998; Pickardt *et al.*, 1999; Pretsch *et al.*, 2005). The packing of the molecules is shown in Fig. 2. Inter­molecular contacts appear normal.

The binuclear molecules lie on the two-fold axes of space group *C2/c*, with the asymmetric unit at 1/2,*y*,1/4. The divalent copper atom, Cu2, shows square-pyramidal coordination, with the four N atoms of the two symmetry-related eten ligands occupying the basal positions, and the N atom of the cyanide group on the two-fold axis in the apical position. The bond length to the apical N atom shows a slight Jahn-Teller extension of 0.10 Å relative to the basal positions (Table 1). The four eten N atoms are roughly co-planar, and the Cu2 atom lies 0.360 (1)Å out of their best plane, in the direction of the apical N atom. The N—C—C—N torsion angle is -54.6 (2)° for each symmetry related chelate ring, giving the ring the λ conformation.

The monovalent copper atom, Cu1, shows trigonal planar coordination to the carbon atoms of the bridging and two terminal cyanide groups, with bond angles C1—Cu1—C2 = 121.33 (5)° and C2—Cu1—C2(1-*x*,*y*,1/2-*z*) = 117.33 (10)°, and Cu1 exactly coplanar with the three cyanide carbon atoms.

The bridging and terminal C—N bond lengths are not significantly different. The bridging C—N group is linearly bonded to the two copper atoms, with the angles Cu1—C—N and C—N—Cu2 both required to be 180° by symmetry. This geometry differs from that found in the one-dimensional polymer [Cu(dien)CN]^+^, (Corfield & Yang, 2012) where both copper atoms are divalent, and the C—N—Cu angle is non-linear at 146.5 (2)°. The Jahn-Teller lengthening of the axial Cu—N distance is greater in the polymer, with Cu—N = 2.340 (3) Å *versus* 2.127 (4) Å in the present structure.

Two symmetry-unique hydrogen bonds link N—H groups from the eten ligand and nitro­gen atoms of terminal cyanide groups from molecules related by translation along the b axis. They are shown in Fig. 3, and details are given in table 2.

### 2.1. Synthesis and crystallization

The compound was prepared by dissolution of 56 mmol of copper(I) cyanide, CuCN, in 30 mL of a solution containing 90 mmol of sodium cyanide, NaCN. To this were added 10 mL of a solution containing 71 mmol of N-ethyl­ethylenedi­amine. Slow evaporation of the deep blue mixture resulted after two days in a yield of 1.87 g of Cu_2_(eten)_2_(CN)_3_ in the form of deep blue thin plates that were often several mm long. The yield for this first batch was 18%, based upon copper.

Total copper was measured iodo­metrically: calculated 33.33%; found 33.23 (4)%, based upon three measurements. The infra-red spectrum, obtained with a Buck Model 530 transmission ir spectrometer, showed two strong CN stretching frequencies at 2,092 cm^-1^ and 2,133 cm^-1^.

### 2.2. Refinement

In the final refinement cycle, the two NH atoms involved in hydrogen bonding were allowed to refine freely. However, atom H3A on N3, which is not involved in hydrogen bonding, was constrained to an ideal position by using a dummy H3B with zero occupancy factor. This dummy atom has been removed from the final coordinates and geometry tables. N—H distances for the refined H atoms were 0.79 (2) and 0.81 (2)Å, shorter than the 0.90Å constrained N—H distance.

### Crystal data

**Table:**

[Cu_2_(CN)_3_(C_4_H_12_N_2_)_2_]	*F*(000) = 788
*M**_r_* = 381.45	*D*_x_ = 1.507 Mg m^−^^3^*D*_m_ = 1.497 (2) Mg m^−^^3^*D*_m_ measured by Flotation in 1,2-dibromopropane/toluene mixtures. Four independent determinations were made. The observed density measurements were systematically 0.7% low, perhaps due to the presence of occlusions in crystals that were large enough to use for density measurements.
Monoclinic, *C*2/*c*	Mo *K*α radiation, λ = 0.71070 Å
*a* = 11.425 (1) Å	Cell parameters from 25 reflections
*b* = 9.679 (2) Å	θ = 5.0–19.1°
*c* = 15.205 (3) Å	µ = 2.53 mm^−^^1^
β = 91.52 (1)°	*T* = 301 K
*V* = 1680.8 (5) Å^3^	Block, dark blue
*Z* = 4	0.33 × 0.30 × 0.30 mm

### Data collection

**Table:**

Enraf–Nonius CAD-4 diffractometer	1674 reflections with *I* > 2σ(*I*)
Radiation source: fine-focus sealed tube	*R*_int_ = 0.020
Graphite monochromator	θ_max_ = 27.0°, θ_min_ = 2.7°
θ/2θ scans	*h* = −14→14
Absorption correction: integration (Busing & Levy, 1957)	*k* = −1→12
*T*_min_ = 0.529, *T*_max_ = 0.587	*l* = −19→19
3737 measured reflections	3 standard reflections every 120 min
1835 independent reflections	intensity decay: 2.3(6)

### Refinement

**Table:**

Refinement on *F*^2^	Secondary atom site location: difference Fourier map
Least-squares matrix: full	Hydrogen site location: inferred from neighbouring sites
*R*[*F*^2^ > 2σ(*F*^2^)] = 0.020	H atoms treated by a mixture of independent and constrained refinement
*wR*(*F*^2^) = 0.062	*w* = 1/[σ^2^(*F*_o_^2^) + (0.*P*)^2^ + 0.250*P*] where *P* = (*F*_o_^2^ + 2*F*_c_^2^)/3
*S* = 1.06	(Δ/σ)_max_ = 0.001
1835 reflections	Δρ_max_ = 0.22 e Å^−^^3^
103 parameters	Δρ_min_ = −0.25 e Å^−^^3^
0 restraints	Extinction correction: *SHELXL97* (Sheldrick, 2008), Fc^*^=kFc[1+0.001xFc^2^λ^3^/sin(2θ)]^-1/4^
Primary atom site location: structure-invariant direct methods	Extinction coefficient: 0.0078 (4)

### Special details

**Table:**

Geometry. All e.s.d.'s (except the e.s.d. in the dihedral angle between two l.s. planes) are estimated using the full covariance matrix. The cell e.s.d.'s are taken into account individually in the estimation of e.s.d.'s in distances, angles and torsion angles; correlations between e.s.d.'s in cell parameters are only used when they are defined by crystal symmetry. An approximate (isotropic) treatment of cell e.s.d.'s is used for estimating e.s.d.'s involving l.s. planes.
Refinement. Refinement of *F*^2^ against ALL reflections. The weighted *R*-factor *wR* and goodness of fit *S* are based on *F*^2^, conventional *R*-factors *R* are based on *F*, with *F* set to zero for negative *F*^2^. The threshold expression of *F*^2^ > σ(*F*^2^) is used only for calculating *R*-factors(gt) *etc*. and is not relevant to the choice of reflections for refinement. *R*-factors based on *F*^2^ are statistically about twice as large as those based on *F*, and *R*- factors based on ALL data will be even larger.

### Fractional atomic coordinates and isotropic or equivalent isotropic displacement parameters (Å^2^)

**Table:**

	*x*	*y*	*z*	*U*_iso_*/*U*_eq_	
Cu1	0.5000	−0.29790 (3)	0.2500	0.04340 (12)	
Cu2	0.5000	0.24064 (2)	0.2500	0.03031 (11)	
C1	0.5000	−0.0984 (3)	0.2500	0.0443 (5)	
N1	0.5000	0.0193 (2)	0.2500	0.0501 (5)	
C2	0.39932 (15)	−0.40213 (16)	0.32653 (12)	0.0440 (4)	
N2	0.34282 (16)	−0.46675 (16)	0.37112 (13)	0.0575 (5)	
N3	0.67206 (12)	0.29094 (15)	0.27009 (10)	0.0403 (3)	
H3A	0.7170	0.2149	0.2662	0.048*	
H3B	0.697 (2)	0.341 (2)	0.2337 (15)	0.061 (7)*	
C4	0.68534 (15)	0.35226 (19)	0.35844 (12)	0.0473 (4)	
H4A	0.6590	0.4475	0.3574	0.071*	
H4B	0.7669	0.3505	0.3778	0.071*	
C5	0.61299 (16)	0.2693 (2)	0.41977 (11)	0.0472 (4)	
H5A	0.6443	0.1765	0.4254	0.071*	
H5B	0.6142	0.3117	0.4776	0.071*	
N6	0.49168 (12)	0.26432 (15)	0.38337 (9)	0.0364 (3)	
H6	0.4663 (16)	0.342 (2)	0.3875 (12)	0.037 (5)*	
C7	0.41570 (17)	0.1642 (2)	0.42882 (12)	0.0498 (4)	
H7A	0.4491	0.0726	0.4232	0.075*	
H7B	0.3394	0.1633	0.3993	0.075*	
C8	0.3996 (2)	0.1943 (3)	0.52557 (13)	0.0670 (6)	
H8A	0.4727	0.1814	0.5571	0.101*	
H8B	0.3420	0.1326	0.5484	0.101*	
H8C	0.3738	0.2880	0.5325	0.101*	

### Atomic displacement parameters (Å^2^)

**Table:**

	*U*^11^	*U*^22^	*U*^33^	*U*^12^	*U*^13^	*U*^23^
Cu1	0.05315 (19)	0.02439 (17)	0.05233 (19)	0.000	−0.00472 (13)	0.000
Cu2	0.03423 (15)	0.02483 (16)	0.03163 (15)	0.000	−0.00377 (10)	0.000
C1	0.0654 (15)	0.0304 (12)	0.0371 (11)	0.000	0.0031 (10)	0.000
N1	0.0788 (16)	0.0256 (10)	0.0459 (12)	0.000	0.0021 (11)	0.000
C2	0.0481 (9)	0.0245 (7)	0.0590 (10)	0.0064 (6)	−0.0022 (8)	−0.0026 (7)
N2	0.0607 (10)	0.0387 (9)	0.0736 (12)	0.0045 (7)	0.0129 (9)	0.0000 (7)
N3	0.0357 (7)	0.0411 (8)	0.0437 (8)	0.0017 (5)	−0.0054 (6)	0.0045 (6)
C4	0.0412 (8)	0.0456 (10)	0.0543 (10)	−0.0025 (7)	−0.0134 (7)	−0.0085 (7)
C5	0.0468 (10)	0.0560 (10)	0.0381 (8)	0.0080 (8)	−0.0121 (7)	−0.0049 (7)
N6	0.0409 (7)	0.0319 (7)	0.0361 (7)	0.0072 (5)	−0.0033 (5)	−0.0012 (5)
C7	0.0598 (11)	0.0481 (10)	0.0418 (9)	−0.0038 (8)	0.0055 (8)	0.0033 (8)
C8	0.0762 (14)	0.0834 (17)	0.0418 (11)	0.0022 (12)	0.0074 (10)	0.0042 (10)

### Geometric parameters (Å, º)

**Table:**

Cu1—C1	1.931 (3)	C4—H4A	0.9700
Cu1—C2^i^	1.9406 (18)	C4—H4B	0.9700
Cu1—C2	1.9406 (18)	C5—N6	1.479 (2)
Cu2—N3	2.0403 (14)	C5—H5A	0.9700
Cu2—N3^i^	2.0403 (14)	C5—H5B	0.9700
Cu2—N6	2.0456 (14)	N6—C7	1.484 (2)
Cu2—N6^i^	2.0456 (14)	N6—H6	0.81 (2)
Cu2—N1	2.142 (2)	C7—C8	1.516 (3)
C1—N1	1.139 (4)	C7—H7A	0.9700
C2—N2	1.136 (2)	C7—H7B	0.9700
N3—C4	1.473 (2)	C8—H8A	0.9600
N3—H3A	0.9000	C8—H8B	0.9600
N3—H3B	0.79 (2)	C8—H8C	0.9600
C4—C5	1.496 (3)		
			
C1—Cu1—C2^i^	121.32 (5)	C5—C4—H4B	110.1
C1—Cu1—C2	121.32 (5)	H4A—C4—H4B	108.4
C2^i^—Cu1—C2	117.35 (9)	N6—C5—C4	108.17 (14)
N3—Cu2—N3^i^	152.39 (8)	N6—C5—H5A	110.1
N3—Cu2—N6	83.96 (6)	C4—C5—H5A	110.1
N3^i^—Cu2—N6	92.97 (6)	N6—C5—H5B	110.1
N3—Cu2—N6^i^	92.97 (6)	C4—C5—H5B	110.1
N3^i^—Cu2—N6^i^	83.96 (6)	H5A—C5—H5B	108.4
N6—Cu2—N6^i^	167.13 (8)	C5—N6—C7	113.62 (14)
N3—Cu2—N1	103.81 (4)	C5—N6—Cu2	107.86 (10)
N3^i^—Cu2—N1	103.81 (4)	C7—N6—Cu2	115.57 (11)
N6—Cu2—N1	96.43 (4)	C5—N6—H6	106.0 (13)
N6^i^—Cu2—N1	96.43 (4)	C7—N6—H6	110.8 (13)
N1—C1—Cu1	180.0	Cu2—N6—H6	102.0 (13)
C1—N1—Cu2	180.0	N6—C7—C8	114.45 (17)
N2—C2—Cu1	177.74 (15)	N6—C7—H7A	108.6
C4—N3—Cu2	107.96 (10)	C8—C7—H7A	108.6
C4—N3—H3A	110.1	N6—C7—H7B	108.6
Cu2—N3—H3A	110.1	C8—C7—H7B	108.6
C4—N3—H3B	111.2 (17)	H7A—C7—H7B	107.6
Cu2—N3—H3B	114.1 (18)	C7—C8—H8A	109.5
H3A—N3—H3B	103.4	C7—C8—H8B	109.5
N3—C4—C5	107.87 (14)	H8A—C8—H8B	109.5
N3—C4—H4A	110.1	C7—C8—H8C	109.5
C5—C4—H4A	110.1	H8A—C8—H8C	109.5
N3—C4—H4B	110.1	H8B—C8—H8C	109.5
			
N3—C4—C5—N6	−54.69 (18)	C5—N6—C7—C8	61.5 (2)

Symmetry code: (i) −*x*+1, *y*, −*z*+1/2.

### Hydrogen-bond geometry (Å, º)

**Table:**

*D*—H···*A*	*D*—H	H···*A*	*D*···*A*	*D*—H···*A*
N3—H3*B*···N2^ii^	0.79 (2)	2.49 (2)	3.181 (2)	147 (2)
N6—H6···N2^iii^	0.81 (2)	2.34 (2)	3.112 (2)	160.9 (17)

Symmetry codes: (ii) −*x*+1, *y*+1, −*z*+1/2; (iii) *x*, *y*+1, *z*.
